# Women self-reported G-spot existence and relation with sexual function and genital perception

**DOI:** 10.4274/tjod.55531

**Published:** 2018-09-03

**Authors:** Aşkı Ellibeş Kaya, Eray Çalışkan

**Affiliations:** 1 Düzce University Faculty of Medicine, Department of Obstetrics and Gynecology, Düzce, Turkey; 2 Bahçeşehir University Faculty of Medicine, Department of Obstetrics and Gynecology, İstanbul, Turkey

**Keywords:** Female Sexual Function index, Grafenberg’s zone, G-spot, vulvar perception

## Abstract

**Objective:**

Aim of study to determine the existence of the G-spot from the healthy women’s point of view and to assess the relationship with sexual function and genital perception.

**Materials and Methods:**

Sexually-active healthy polyclinic patients aged between 18 and 54 years (n=309) were classified into three groups as group 1 (do not agree, n=90, 29.1%), group 2 (neutral/do not know, n=61, 19.7%) and group 3 (agree, n=158, 51.1%) with regard to participants’ responses to a question of “does the G-spot exist.” The Female Sexual Function index (FSFI) and Female Genital Self-Image scale (FGSIS) were administered to the participants.

**Results:**

Half of the patients (51.1%, n=151) indicated that the G-spot exists. The groups were statistically homogeneous in terms of body mass index, parity, marital status, number of partners, and sexual orientation (p=0.41, p=0.06, p=0.12, p=0.19, p=0.25; respectively). Women with an education level of “less than high school” reported the absence of the G-spot significantly more often than others, whereas women with an education level of “university and higher” reported the presence of the G-spot more often (p≤0.001). Sexual dysfunction was found to be more frequent in group 1 when compared with group 3 (p=0.002, 67.8%, 45.6%). The orgasm subdomain scores of the FSFI and FGSIS total scores were significantly higher in group 3 than in group 1 (p<0.001, p=0.041).

**Conclusion:**

Half of healthy women in the Turkish population believe that the G-spot exists. Those women showed better scores in sexual functioning and genital perception.

**PRECIS:** Women self-reported G-spot existence.

## Introduction

Female sexuality is complex and is influenced by many factors related to physiologic, psychological, hormonal, social, cultural, and partner issues. The vital organ in males is the penis, whereas the uterus, vagina, clitoris, and the Grafenberg-spot (G-spot), whose existence is not definite, are among the factors that are effective in women^(1)^. The G-spot is a current and controversial issue, and it now attracts interest in female sexuality because it involves a market share in genital esthetics with interventions such as its augmentation^(2)^. Ernst Grafenberg was the first to describe the G-spot as an erogenous zone approximately half a centimeter in size, below the urethra on the anterior wall of the vagina, but the first reports of its presence date back much further. During orgasm, this area is pressed downwards like a small cystocele protruding into the vaginal canal^(3)^. The G-spot was named after Addiego’s case report on female ejaculation thirty years later^(4)^. In this article, it was stated that when a 1.5-2 cm area extending along the long axis of the urethra was touched, it gave rise to a urination feeling, and when stimulation was sustained, it was stimulated in sexual terms, and with this stimulation, the area was enlarged at a rate of 50%. About the same subject, anatomists, gynecologists, and sexual experts published self-reported questionnaire studies, case studies, anatomic and histologic studies and imaging studies^(5,6,7,8,9,10,11,12,13)^. Whether it is really anatomically present or a scientific deception still awaits an answer and publications are contradictory^(14,15)^. In this study, we asked in detail whether the participant felt a coin-size sensitive area in the anterior vaginal wall at the time of finger or penis penetration or pressure; namely the G-spot. The purpose of the study was to investigate how many women who as owners of this zone and to investigate its possible effect on sexual function and female genital perception.

## Materials and Methods

The institutional Ethics Committee approved the study (Düzce University) (approval number: 2018/81), and written informed consent was obtained from all individual participants included in this study.

The descriptive cross-sectional study was conducted in a medical faculty between January 2018 and April 2018. The questionnaires were administered to healthy female participants who reported no known illnesses. Sexually-active and premenopausal patients ageg over 18 years who were admitted to our hospital polyclinic for routine gynecologic examinations were admitted to the study. Patients with esthetic concerns, those planning to undergo genital esthetic procedures, postmenopausal patients, those who had never had vaginal sexual intercourse, incontinence, pelvic organ prolapses, menstrual disorders and gynecologic cancer history, gynecologic surgery for any reason, oral contraceptive and antidepressant medication, with episiotomy, and those using intrauterine devices were excluded from the study. A total of 309 participants who agreed to participate in the study, who met these criteria, and who completed the questionnaire were included in the analyses of the study. Two lesbian participants were excluded from the study because they said they had not experienced any vaginal sexual intercourse, and five lesbian participants were included in the study because they stated that they engaged in vaginal sexual intercourse. The participants were taken into a quiet room, and their demographic data were recorded, the questions on the G-spot were asked, and the Female Sexual Function index (FSFI) and Female Genital Self-Image scale (FGSIS) questionnaires, which have been validated for the Turkish language, were administered^(16,17)^. The FSFI is a brief instrument for the assessment of sexual function consisting of 19 questions. It was validated based on the Diagnostic and Statistical Manual of Mental Disorders-Fourth Edition (diagnoses of desire disorder, arousal disorder, and orgasmic dysfunction). Questions are scored for domains of libido, arousal, lubrication, orgasm, satisfaction, and pain^(18)^. Female sexual dysfunction was defined as a total score of 26 or less (maximum possible score of 36)^(19)^. To investigate the relationship between the G-spot and orgasm, the orgasm subdomain scores were also calculated (for orgasm subdomain, a maximum possible score of 6). Based on the fact that female sexual functions are associated with genital perception, we applied the FGSIS, which has been validated for the Turkish language^(17)^. The FGSIS is a 7-item questionnaire and is easy to apply, and shows female genital perception^(20)^. The question related to the G-spot asked was as follow: “Is there a region on the front of your vagina where you urinate and where you feel more sensitive when stimulated with a finger or penis during sexual intercourse?; Answers were collected in the form of “No, I do not agree,” “I am undecided-I do not know,” “Yes, I agree.” According to the responses given to the G-spot questions, the participants were divided into groups 1, 2, 3; and then, the analyses were made.

### Statistical Analysis

Continuous data are summarized as mean ± standard deviation and categorical data as frequency and percentage. The independent Samples t-test and One-Way analysis of variance were used to compare groups. Relations between categorical variables were examined using Pearson’s chi-square or Fisher’s exact tests. When significant results were found, subgroup analyses were performed with Bonferroni correction. Correlations between continuous data were analyzed using Pearson’s correlation coefficient. The IBM SPSS Statistics for Windows, Version 22.0. Armonk, NY: IBM Corp. (IBM, SPSS Inc., USA) statistical software package was used, and the significance level (p) was considered as <0.05.

## Results

The demographic data of the patients are given in Table 1. Among all participants, there were 151 (51.1%) participants who said that the G-spot existed; 90 (29.1%) participants said “No, there is no such region” and 61 (19.7%) participants said that they were indecisive or did not know. The mean age was 35.8±5.9 (minimum-maximum, 18-54) years. When the age groups were divided into categories as 18-24, 25-34, 35-44, 45-54 years, there were statistically significant differences between these age groups (p=0.03). In the subgroup analysis performed with Bonferroni correction, it was seen that the significant difference was only in the 45-54 age group, in group 2 and group 3. Regarding body mass index, parity, marital status, partner count, and sexual orientation, no statistically significant differences were detected between the G-spot groups (p=0.41, p=0.06, p=0.12, p=0.19, p=0.25). There were significant differences regarding levels of education (p≤0.001). It was observed that the university and more education group stated that the G-spot existed at a higher rate, and the group that consisted of high school and below levels of education stated that the G-spot did not exist at a higher rate. The FSFI and FGSIS comparative analyses with G-spot groups are shown in Table 2. FSFI total score averages were found as 21.0±8.9; 22.8±8.5; 24.8±8.6, respectively, according to the groups that did not agree with the existence of the G-spot, and those that were indecisive, and agreed. The total scores were statistically different between the groups (p=0.004). There was a significant difference (p=0.002) between the G-spot groups when the FSFI total score was divided into the two groups as those scoring below and above 26 (p=0.002). According to the post-hoc test result, this difference was found between group 1 and 3. It was also determined that those who were indecisive showed similarities in both groups. In terms of the FSFI orgasm subdomain, there were significant differences between the groups according to the G-spot agreement status (p<0.001); this difference was found between all groups according to the post-hoc test result. We also found that there was a significant difference between the groups in terms of FGSIS total scores (p=0.041). According to the post-hoc test result, this difference was between those who said that there was a G-spot and those who said that there was no such spot. It was also determined that those who were indecisive showed similarities between the groups.

The FGSIS score was positively correlated at a weak level with both the FSFI (r=0.277, p<0.001) and the FSFI-orgasm (r=0.282; p<0.001).

## Discussion

Although the existence of the G-spot is usually accepted by the general public, it is anatomically controversial^(5,7,14,21,22)^. Biopsy studies showed that the anterior vaginal wall was more densely innervated than the posterior, and the distal region contained a higher number of nerve fibers than the proximal region^(23)^. Microdissection and immunohistochemical studies with seven fresh cadavers confirmed the abovementioned data and that the distal anterior vaginal wall was thicker than the proximal wall^(24)^. Although these data reveal the more sensitive and evident open-to-stimuli structure of the anterior vaginal wall, there are biopsy results showing the opposite viewpoint^(5,7)^. Ostrenzky first anatomically identified the neurovascular structure that he called the G-spot complex in a fresh cadaver in 2012^(6)^. Two years later, the same author published a cadaver study in which the histology of the G-spot complex was shown. In this study, the G-spot complex was detected anatomically in all eight cadavers, and the tissues were shown histologically by staining with hematoxylin and eosin in two randomized cases^(22)^. In a recent article by Hoag et al.,^(7)^ which described the most extensive anatomic study of the anterior vaginal wall containing a detailed dissection of thirteen cadavers, the G-spot could not be described in the front wall of the vagina. In these two separate cadaver studies, it is confusing that the G-spot was defined at a rate of one hundred percent in one study yet the other study could not define it at a rate of one hundred percent. Studies are contradictory. In a study conducted by Puppo and Gruenwald^(2)^, Puppo and Puppo^(25)^ in which they reviewed the terminology of female sexuality, they wrote that the G-spot did not exist under the subtitle of “The G-spot does not exist: Is it a scientific fraud?” They stated that there was no vaginal orgasm and added that there was no scientific support for research that said the G-spot and vaginal orgasm existed. It was stated in some previous reserach that perhaps the G-spot was formed with a pudendal nerve innervation in areas that varied from person to person in the front side of the vagina instead of a same specific area in everybody^(1)^. In a previous study that was conducted by asking questions to patients, there were 1234 participants in the first large-scale G-spot self-reported questionnaire study. In this study, the G-spot identification rates were determined as high as 84.3%. It was determined that approximately 3 years after the age of the first relationship, people were found to have reached orgasm through sexual intercourse, and they discovered the sensitive region of the vagina about 6 years after the first orgasm^(11)^. One year later, the self-reported G-spot study of the same group, which included 1289 patients, revealed a G-spot detection rate of 82%. In this study, however, the sampling consisted of nurses, sex therapists, and counselors who had a high-level of education and who were very familiar with these issues^(12)^. The high rates may be due to the nature of the sampling. In another self-reported questionnaire that was recently published, the rate of expressing G-spot existence was 56%. In this study conducted on twins to investigate the genetic basis of G-spot existence, no genetic basis was found. The reason for this may be that people cannot discover their G-spot via environmental factors^(10)^. In this study, the oldest patient was aged 83 years, and the rate of elderly participants who stated that the G-spot existed was lower. This result is not surprising. In our study, postmenopausal participants were excluded from the study and it was seen that the value that was found to be significant was in the 45-54 years’ age group, and in the group that was indecisive, which we considered having no clinical significance. In our study, the rate of participants who believed in the existence of the G-spot was found as 51.1%. This ratio is compatible with the literature^(10)^. The percentage of those who thought that there was a G-spot in the university graduate group was high. In a similar study, there was no difference between the levels of education and the responses^(10)^. In our study, the difference between the levels of education and the G-spot groups could be due to the change in the understandability of the problem with education. Another possibility is that college graduates who use the right resources for accessing information more accurately can have higher G-spot awareness or greater exposure to media. People may have discovered their bodies better by reading and practicing what they read. Filling materials such as hyaluronic acid, autologous oil injections, and G-spot augmentations are rapidly increasing worldwide today. In a case report with autologous fat injection, no change was determined in the sexual function questionnaire before and after the application and there was no increase in experiencing orgasms^(26)^. The vagina is a dynamic organ that plays an active role in sexual intercourse. Anatomic relationships and dynamic interactions between the clitoris, urethra, and anterior vaginal wall have led to the concept of a clitourethrovaginal complex, which defines a versatile, functional area that may induce orgasmic responses when properly stimulated during penetration^(27)^. It is emphasized that this means a broader meaning beyond a spot. In another study, a strong and reverse relation was found between the distance of a woman’s clitoris and her urethral meatus. It was emphasized that this result was secondary to more stimuli due to the increased pressure on the vaginal wall and nerve extensions of the clitoris into the vagina^(28)^. On the same subject, another study under the title of “echography of the G-Spot” measured the urethrovaginal space thickness using introital ultrasonography, and the association with vaginal orgasm was examined; it was found that this measurement was directly related to vaginal orgasm^(29)^. Those who think that G-spot exists have higher genital perception and sexual function scores compared with other participants. In light of the above studies, it is not wrong to claim that women are more vaginally stimulated when they feel that there is a G-spot. For this reason, it is not surprising that these women’s sexual functions, especially orgasm subdomains, are high. In this group, another reason that the genital perception scores may have been perceived as high might be due to the fact that the sexual functions were good in this group, higher than the group that claimed that there was no G-spot. In our study, there was a positive correlation between FGSIS and FSFI, as it was in the original study of the genital perception questionnaire. It is known that the self-image of the person affects sexual functions, which proves this^(20)^. The FGSIS has a positive correlation with all subdomains except the desire domain of FSFI^(30)^. In our study, the weak positive correlation could be attributed to the multifactorial nature of female sexuality.

A person’s exploration of sexuality is a process, and the fact that they do not know the sensitive areas of the vagina may mean that such areas do not exist in reality as well as that one has not yet discovered these areas. This can prolong this process in countries where sexuality is a taboo subject of discussion, where experiences before marriage are few and the possibility of having sexual experience with different partners before marriage is low. Partner incompatibility is another factor in this subject. For this reason, the fact that the participants do not know the existence of the G-spot or are indecisive about its existence does not mean that this point does not actually exist in reality. The fact that the present study was self-reported and the sample being small are limitations. The possibility of not understanding the question is another limitation. In further investigations, in addition to the self-reported questionnaires in wider series, the aim is to determine the location of the G-spot with a finger during an examination and compare the self-reported answers with the examination findings.

G-spot presence continues to be an interesting subject in the academic environment and for the public. The biggest reason for this might be that there is no consensus on its existence. This issue will continue to attract interest until definitive and descriptive studies are made.

## Conclusion

Half of the participants stated that G-spot existed, which was consistent with the literature. An increase in sexual function, orgasm scores, and genital perception scores of these women was identified. Self-reported questionnaires give an idea of ​​G-spot existence but are inadequate as proof. Further histologic and anatomic studies are needed with larger series.

## Figures and Tables

**Table 1:**
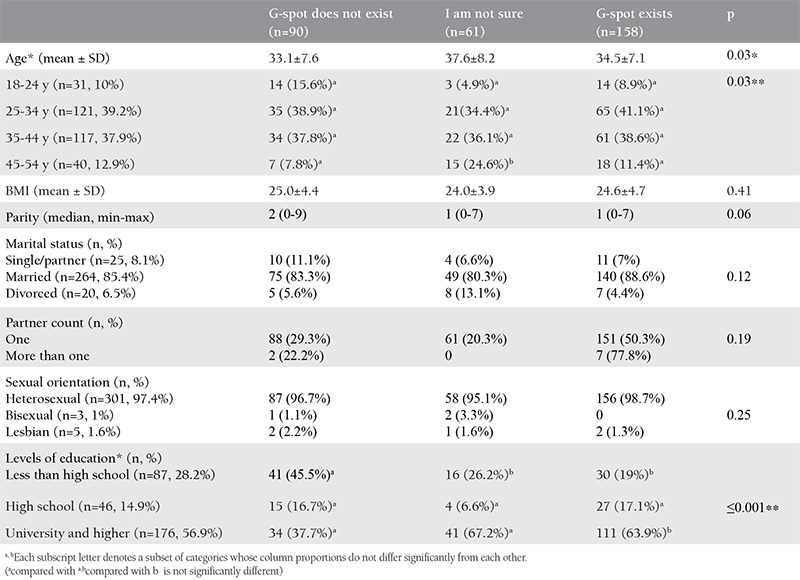
Self-reported G-spot existence among groups and demographic data

**Table 2:**
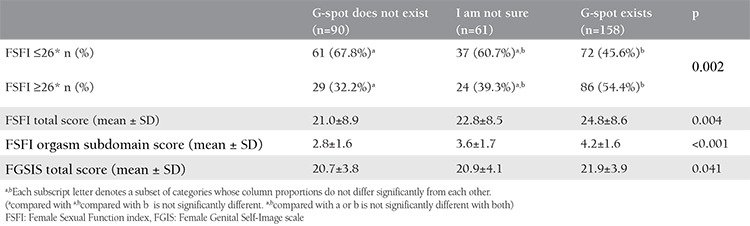
Patients’ self-reported G-spot existence and relation with Female Sexual Function index and Female Genital Self-Image scale
